# Delayed Amplatzer Occluder Device Closure of Postinfarction Ventricular Septal Defect: A Case Report

**DOI:** 10.1155/2014/159010

**Published:** 2014-03-03

**Authors:** Francis Ting, Aditya Bhat, Neville Sammel, David Muller

**Affiliations:** ^1^St Vincent's Hospital Sydney, NSW 2010, Australia; ^2^Blacktown Hospital Sydney, NSW 2148, Australia

## Abstract

Postinfarction ventricular septal defect (VSD) is a rare complication after acute myocardial infarction, with an incidence rate of 1-2% of all myocardial infarcts (Hutchins, 1979). It is a medical emergency with sobering survival numbers, having a mortality rate of 70–80% within two weeks of the incident event (Bouchart et al., 1998). Cardiac surgery is considered the gold standard in the management of these defects; however, its main limitation is that it carries a high risk of perioperative mortality and postoperative sequelae. Percutaneous transcatheter closure of VSD is a relatively new method of repair. Due to scarcity of reports in the literature, there is limited data regarding survival data; however, noninferiority to surgery has been demonstrated in one case series (Papalexopoulou et al., 2013). Long-term follow-up studies are lacking, and thus long-term mortality has yet to be discerned. We present a case of an 87-year-old female who, following postmyocardial infarction VSD, developed clinically significant heart failure. The patient was reluctant to undergo open repair given her age and comorbidities and she underwent successful percutaneous repair of her VSD using a 16 mm Amplatzer occluder device 18 months after her initial presentation.

## 1. Introduction

Percutaneous transcatheter closure of ventricular septal defect (VSD) is a relatively new technique. Particularly, in patients precluded from open cardiac surgery given their age or comorbidities, percutaneous closure is a viable option. We present a case report of an Amplatzer device closure of postinfarct VSD in a clinically contextual manner highlighting considerations of patient age and comorbidities, type of presentation, timing of procedure, and also device sizing.

## 2. Case Report

An 87-year-old woman presented to a tertiary referral hospital with progressive dyspnoea and decreased exercise tolerance following a fall. She denied chest pain, palpitations, or syncope. She had a past history of hypertension, hypercholesterolemia, and impaired glucose tolerance.

Physical examination revealed a regular pulse of 110 beats per minute. Her supine blood pressure was 120/50 mmHg and there was a harsh grade III holosystolic murmur heard best at the apex. She had clinical and radiological signs of congestive cardiac failure.

The 12-lead electrocardiograph (ECG) revealed sinus tachycardia (110 beats per minute), with 2 mm ST elevation in the anteroseptal leads (*V*_2_–*V*_4_) and a left anterior fascicular block. Serum troponin T level at admission was elevated (163 ng/L).

**Figure 1:**
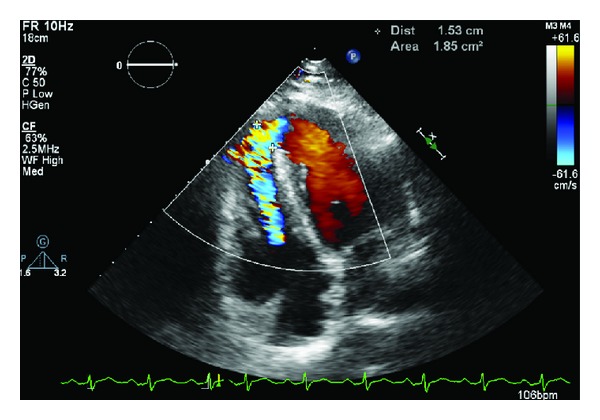
Transthoracic echocardiogram: apical ventricular septal defect with shunt noted.

**Figure 2:**
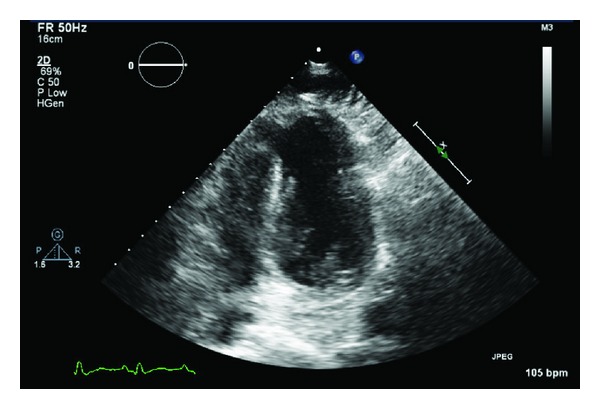
Transthoracic echocardiogram demonstrating ventricular septal defect.

Transthoracic echocardiography (Figures 1 and 2) revealed apical hypokinesis and an apical muscular ventricular septal defect (VSD) measuring approximately 1.6 cm in diameter. She had features of moderate pulmonary hypertension, with an estimated pulmonary artery systolic pressure of 42 mmHg. Left ventricular wall thickness was within normal limits and there was no atrial dilatation. Left ventricular function was within normal limits with an estimated ejection fraction of 65%. She had a left to right shunt with *Q*_*p*_/*Q*_*s*_ ratio of 1 : 1.8.

Technetium (99mTc) sestamibi myocardial perfusion scanning revealed a small apical nontransmural infarct, with small area of peri-infarctional ischaemia. She was diagnosed as having an acute apical myocardial infarction complicated by ventricular septal defect.

In view of her age, iron deficiency anaemia due to haemorrhoidal bleeding, and the absence of ongoing angina, it was decided that she should not be anticoagulated or referred for diagnostic coronary angiography. She was also considered unsuitable for cardiac surgery.

Following discharge from hospital, patient complained of lethargy and breathlessness and developed a recurrence of cardiac failure. In view of her age and comorbidities as well as a reluctance to undergo open repair, the decision was made to perform percutaneous closure of the VSD. This was performed in delayed fashion 18 months after the patient's initial presentation, the rationale being that this would allow the edges of the defect to fibrose to optimise the landing zone and to let the patient stabilise from her recent hospital admissions.

**Figure 3:**
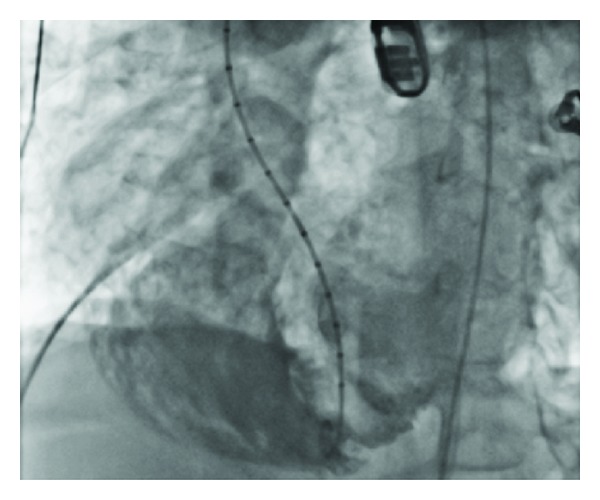
Left ventriculography: shunt of contrast noted.

**Figure 4:**
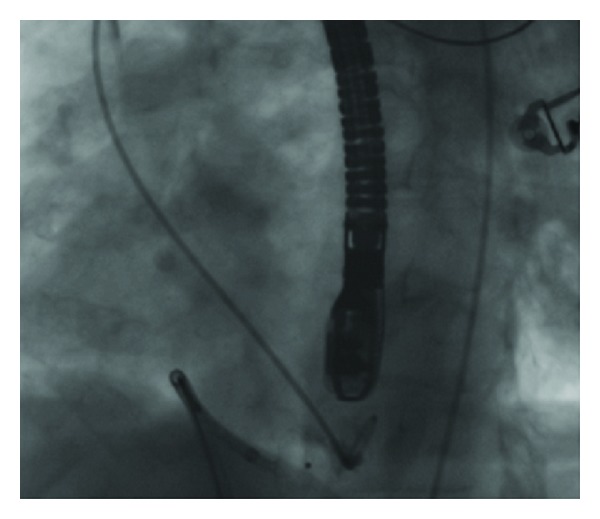
Percutaneous transcatheter Amplatzer occluder device inserted across apical ventricular septal defect.

A 16 mm Amplatzer muscular VSD occlude device was deployed (Figures 3 and 4), with transesophageal echocardiography confirming good placement of the device with reduction in the degree of shunting. Left ventriculography demonstrated a small residual flow around the apical side of the device but this appeared to reduce with time. It was felt that device sizing was appropriate and this residual shunting was related to the irregular shape of the postinfarction defect and is likely to lessen with time as the disks become endothelialised.

The patient subsequently made a successful postprocedural recovery and is clinically well to date.

## 3. Discussion

Postinfarction VSD is a rare complication after acute myocardial infarction, with an incidence rate of 1-2% of all myocardial infarcts [1]. It is a medical emergency with sobering survival numbers, having a mortality rate of 70–80% within two weeks of the incident event [2].

Infarction associated with septal rupture is usually transmural and quite extensive, with anatomical location of the infarct primarily occurring within the anterior wall (60%) [3]. Primary complications include papillary muscle rupture leading to mitral insufficiency, periventricular aneurysm, and acute decompensated cardiac failure [4]. Furthermore, there is great risk of expansion of the defect secondary to the friability of necrotic myocardium, as well as exposure to shear stress of septal branches early after VSD occurrence [5, 6]. These factors lead to an impairment of left ventricular function with compromise of organ perfusion, leading to cardiogenic shock and death. Therefore, emergent intervention is commonly required.

Cardiac surgery is considered the gold standard in the management of endocardial cushion defects, including acquired rupture, with the first surgical repair being performed in the late 1950s [7]. A statistical mortality benefit relative to medical management has been demonstrated, with case series reporting early mortality of 46.0 ± 5.0% [8]. Despite modest improvements in survival, surgical intervention has its limitations.

Firstly, there is a high risk of perioperative mortality and postoperative sequelae. Haemodynamic deterioration during surgery is a strong predictor of early mortality, greater than that of cardiogenic shock on admission [8]. Secondly, temporal limitations arise, with intervention becoming restricted to VSD closure in the subacute and chronic settings. Some cardiothoracic surgeons recommend a 6-week waiting period for adequate collagen deposition on tissue edges to allow for adequate apposition and anchorage of the surgical patch during operation; however, advances in surgical technique have resulted in a move towards early surgical repair in selected cases [2, 9].

Given the above, alternative methods of repair have been sought. Percutaneous transcatheter closure of VSD is a relatively new method of establishing separation of pulmonary and systemic circulations and thus reducing transseptal flow. Due to scarcity of reports in the literature, there is limited data regarding survival data; however, the few reported series have shown an overall mortality rate of 44–60% within one year [10]. Furthermore, noninferiority to surgery has been demonstrated in one case series [10]. Long-term follow-up studies are lacking, and thus long-term mortality has yet to be discerned.

The Amplatzer postinfarct muscular VSD device is a self-centering nitinol occluder that is comprised of two discs connected by a waist. It is used to cover the defect, with occlusion occurring primarily via in situ thrombosis of its waist. It contains a retention skirt which conforms to the adult muscular septum which helps secure the device in place. Its ability to self-center allows for several attempts at positioning due to ease of retrieval prior to release. Furthermore, given that occlusion is achieved via the central portion of the occluder, it is less affected by septal wall irregularities. The self-expansile properties of the device are helpful in the management of defect enlargement [11].

In this case study, a residual postoperative shunt was present. This finding is reflected in the literature, with case series reporting a high incidence of residual postoperative shunting [11]. Reasons for this complication result from the structural irregularity of myocardial necrosis and apical dislocation of occlude devices. Furthermore, due to myocardial friability, implantation carries a risk of extending ventricular rupture and further impeding interventricular cardiac dynamics.

This is, to our knowledge, the first documented Amplatzer device closure of a postinfarct VSD in Australia. The use of transcatheter closure is an emerging modality and in selected patients, it is a useful alternative to surgical intervention in acquired VSDs. However, further long-term data needs to be acquired to further assess the long-term viability of this intervention.
